# Traditional Chinese medicine injection for the treatment of viral pneumonia in children: A protocol for systematic review and meta-analysis

**DOI:** 10.1097/MD.0000000000025506

**Published:** 2021-04-23

**Authors:** Li Fang, Jiaoru Pei, Song Mao, Liangxia Wu, Siqiong Jiang

**Affiliations:** Department of Pediatrics, Shanghai Sixth People's Hospital, Shanghai, China.

**Keywords:** children, viral pneumonia, systematic review, traditional Chinese medicine injection

## Abstract

**Background:**

In recent years, more and more reports are focused on the application of traditional Chinese medicine injection (TCMJ) for the treatment of viral pneumonia. There are about 200 million cases of viral pneumonia worldwide every year, half of which are children. At present, many kinds of TCMJ are created for the treatment of viral pneumonia in children, with good therapeutic effects. However, there are many kinds of TCMJ, and the treatment advantages are different, thus bringing difficulties to the selection of clinical drugs. In order to provide evidence-based evidence support for the clinical selection of TCMJ for the treatment of viral pneumonia in children, this study selected the commonly used TCMJ for clinical treatment of viral pneumonia for meta-analysis to evaluate its efficacy.

**Methods:**

The Chinese Biomedical Literature Database, China National Knowledge Infrastructure, Wanfang Data, Viper information databases, Cochran library Web of Science, PubMed, MEDLINE and EMBASE will be searched. The literature will be searched, with language restriction in English and Chinese. The related reference will be retrieved as well. Two reviewers will independently extract data and perform quality assessment of included studies. Review Manager 5.3 will be applied to conduct this meta-analysis.

**Results:**

The results of this systematic review and meta-analysis will be published in a peer-reviewed journal once we finish this study.

**Conclusions:**

This study provides reliable evidence-based evidence for the efficacy of TCMJ in the treatment of viral pneumonia in children.

**Ethics and dissemination:**

We will not be allowed to publish private information from individuals. This kind of systematic review should not harm the rights of participants. No ethical approval was required. The results can be published in peer-reviewed journals or at relevant conferences.

**OSF Registration number:**

DOI 10.17605/OSF.IO/795MB.

## 1 Introduction

Viral pneumonia in children is one of the common diseases in pediatrics, and it is commonly caused by respiratory syncytial virus, influenza virus, adenovirus and so on.^[1–3]^ It is easy for these viruses to damage the respiratory system, and to invade the circulatory system and central nervous system. If it is not controlled in time, it can cause serious damage to the body.^[4]^ The disease has two characteristics, namely rapid onset and rapid progression. Most of the clinical manifestations include fever, cough, expectoration, wheezing and other symptoms.^[5,6]^ In severe cases, it can cause respiratory failure, and then endanger children's live.^[7]^ At present, the incidence of the disease is gradually increasing.^[8]^ It cannot be a targeted treatment,^[9]^ because there are many kinds of pathogenic viruses, frequent variation and easily produced drug resistance.

Western medicine is mainly treated with antiviral drugs, including ribavirin, acyclovir, interferon and adenosine arabinoside, but a unified treatment plan has not yet been formed.^[10]^ However, due to the large variety of viruses and frequent variation, it is not difficult to produce drug resistance, poor efficacy, and other problems.^[9,11]^

Viral pneumonia belongs to the category of “wind-warming and lung-heat disease” in traditional Chinese medicine (TCM).^[12]^ Although the pathogens are different, there are similarities in the mechanism, clinical manifestation and treatment of the disease that is caused by the virus.^[13]^TCM treatment should clear heat and detoxify, release the lung and dissipate phlegm.^[14,15]^ Studies have revealed that traditional Chinese medicine injection (TCMJ) such as Xiyanping, Tanreqing and Reduning, has obvious advantages and characteristics in terms of the prevention and treatment of infantile viral pneumonia.^[7,16]^ However, the homogenization of these TCMJ is serious, and the efficacy and indications are similar, but the difference in curative effect is not clear, so doctors and patients are at a loss when choosing drugs. The purpose of this study is to systematically evaluate the efficacy of TCMJ in the treatment of viral pneumonia in children by adopting the principles and methods of evidence-based medicine. At the same time, it also provides some reference materials for the treatment plan of coronavirus disease 2019 for children.

## 2 Methods

### 2.1 Registration

The protocol followed the guideline of the Preferred Reporting Items for Systematic Review and Meta-Analysis Protocols.^[17]^ Moreover, it has been registered on Open Science Framework (Registration number: DOI 10.17605/OSF.IO/795MB).

### 2.2 Patients and public involvement

No patients involved.

### 2.3 Searching strategy

The Chinese Biomedical Literature Database, China National Knowledge Infrastructure, Wanfang Data, Viper information databases, Cochran library Web of Science, PubMed, MEDLINE and EMBASE databases will be searched from inception time to date. Studies concerning the effects of TCMJ in the treatment of viral pneumonia in children will be included in this meta-analysis, and randomized controlled trials (RCTs). The related reference will be retrieved as well. The literature will be searched, with language restriction in English and Chinese. The detailed information of PubMed search strategy is presented in Table 1.

**Table 1:** Search strategy in PubMed database.

Number	Search terms
#1	Pneumonia, Viral[MeSH]
#2	Pneumonias, Viral[Title/Abstract]
#3	Viral Pneumonia[Title/Abstract]
#4	Viral Pneumonias[Title/Abstract]
#5	#1 OR #2 OR #3 OR #4
#6	Chinese herbal injections[Title/Abstract]
#7	Traditional chinese medicine injections[Title/Abstract]
#8	Traditional chinese medicine[Title/Abstract]
#9	#6 OR #7 OR #8
#10	#5 AND #9
#11	Random∗[Title/Abstract]
#12	#10 AND #11

### 2.4 Eligibility criteria

RCTs will be included in this meta-analysis.

### 2.5 Exclusion criteria

1) Incomplete data or misrepresentation of data reports; 2) repeated publication of documents; 3) case reports, reviews, etc.; 4) inability to obtain original documents.

### 2.6 Participants

Children enrolled should be diagnosed as virus pneumonia.

### 2.7 Interventions

The experimental group was treated with both control group treatment and TCMJ, and the type of TCMJ was not limited. The control group was standard treatment (e.g. ribavirin, ganciclovir, interferon and so forth).

### 2.8 Outcomes

Main outcomes: Effective rate.

Additional outcomes: 1) Time for body temperature to return to normal; 2) Cough disappearance time; 3) Rale disappearance time; 4) Hospitalization time; 5)Body temperature began to drop time; 6) Antiasthmatic time; 7) X-ray inflammation absorption time; 8) Adverse reactions.

### 2.9 Study selection and data extraction

#### 2.9.1 Study selection

**Figure 1:**
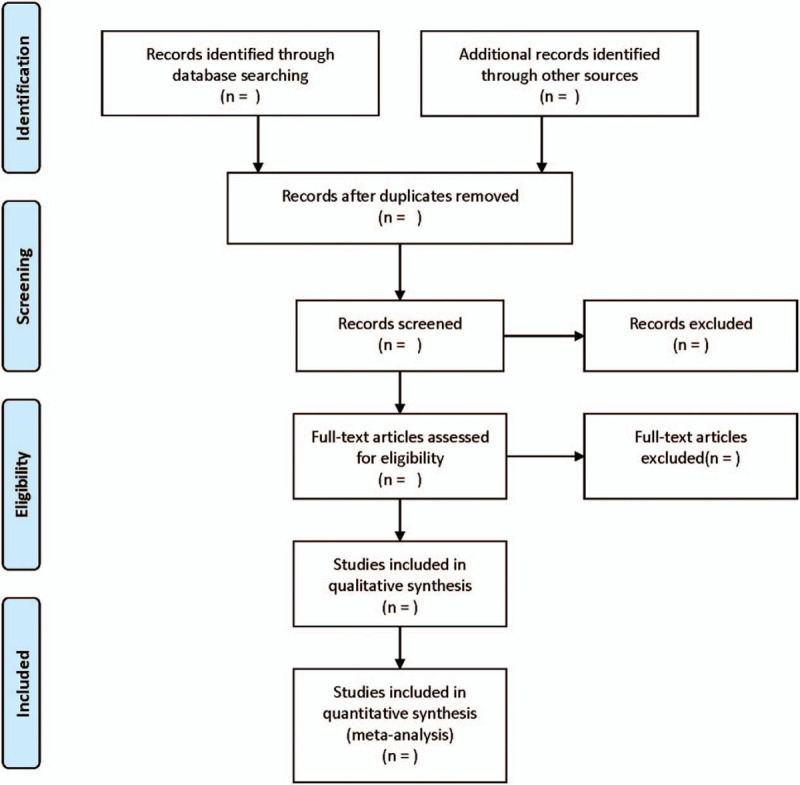
The process of literature filtering.

Two reviewers independently select studies, and any disagreement between the two reviewers should be consulted by a third reviewer for a consensus. We will remove repetitive articles at first and exclude irrelevant studies based on the title, abstract and the full text. The study selection process is displayed in a Preferred Reporting Items for Systematic Review and Meta-Analysis Protocols flow diagram (Figure 1).

#### 2.9.2 Data extraction

A standardized form will be used by two reviewers to extract data independently, and disagreements between them should be solved with the help of a third reviewer. The detailed extraction information is as follows: the first author, publication date, country, sample size, age, type of TCMJ, content and duration, intervention time, intervention details, etc.

### 2.10 Quality assessment of included studies

The Cochrane Systematic Review Manual recommended bias risk assessment method for risk assessment for inclusion in RCT. The main items are as follows: 1) Randomization plan; 2) Group concealment; 3) Blind method; 4) Incomplete data reporting; 5) Selective outcome report; 6) Other sources of bias. Each item is evaluated as “high”, “low” or “unclear”.

### 2.11 Data synthesis and statistical analysis

#### 2.11.1 Data synthesis

Review Manager 5.3 (The Cochrane Collaboration, Software Update, Oxford, UK)) will be used to conduct this meta-analysis. The standardized mean difference with 95% confidence intervals (95% CIs) is applied to calculate continuous variables. The relative risk ratio with 95% CIs is used to calculate binary variables.

#### 2.11.2 Assessment of heterogeneity

Statistical heterogeneity among included studies will be assessed by carrying out the χ2 test and I2 test. We will adopt a fixed-effect model for data analysis at first. If I2 > 0.5 or p < 0.1, it is considered that there is a significant heterogeneity among the studies, and random-effect model will be accepted without examining the probable cause for the high heterogeneity.

#### 2.11.3 Subgroup analysis

Subgroup analyses will be performed on the type of TCMJ and the efficacy of TCMJ.

#### 2.11.4 Sensitivity analysis

Sensitivity analysis is conducted by excluding studies one by one, so we can determine the source of heterogeneity.

#### 2.11.5 Assessment of publication bias

Egger's test and Begg's test will be conducted to quantitatively assess the publication bias.^[18]^

### 2.12 Ethics and dissemination

Due to the nature of this meta-analysis, ethical approval is not required, which is based on published papers. The results of this systematic review and meta-analysis will be published in a peer-reviewed journal once we finish this study.

## 3 Discussion

Viral pneumonia is a disease characterized by pulmonary dysfunction, and it is caused by the downward spread of the virus and the inflammation of the lung parenchyma. In recent years, the incidence of viral pneumonia has gradually increased, especially in infants under 2 years old.^[19]^ At present, the treatment of viral pneumonia in children is not unified. Western medicine is often applied to treat this disease with antiviral drugs such as ribavirin, but it is obvious that the therapeutic effects of these drugs are not satisfactory, and there are many adverse reactions.

TCM treatment is generally based on clearing lung and detoxification, and relieving cough and asthma. TCMJ has been widely used in clinical practices in China, and it is widely used by patients suffering from respiratory diseases.^[20]^ In China, the effectiveness of TCM in the control of infectious diseases has been proved. Therefore, the Chinese government encourages the application of TCM to fight viral pneumonia.^[21]^ Xiyanping injection, Xuebijing injection, Reduning injection and Tanreqing injection can improve the total clinical effective rate, shorten the duration of main clinical symptoms, and sign in the treatment of viral pneumonia.^[22–27]^ However, in many countries in the world, TCMJ for the treatment of viral pneumonia in children is still challenging.

At present, the clinical trial data on the efficacy and safety of TCMJ for the treatment of viral pneumonia in children are limited, and there is insufficient comprehensive review and summary. Therefore, the purpose of this paper is to explore the efficacy of drug injection in the treatment of viral pneumonia in children, and to provide decision-making basis for clinical diagnosis and treatment of viral pneumonia in children. As far as we know, this is the first systematic review and meta-analysis of the efficacy of TCMJ for the treatment of viral pneumonia in children.

## Acknowledgments

This work is supported by the Scientific Research Project of Shanghai Health Bureau(20174341).

## Author contributions

**Conceptualization:** siqiong jiang.

**Data collection:** Li Fang and Jiaoru Pei.

**Data curation:** Li Fang, Jiaoru Pei.

**Formal analysis:** Li Fang.

**Funding acquisition:** siqiong jiang.

**Funding support:** Siqiong Jiang.

**Literature retrieval:** Song Mao.

**Project administration:** siqiong jiang.

**Resources:** Jiaoru Pei.

**Software operating:** Liangxia Wu.

**Software:** Jiaoru Pei, Song Mao.

**Supervision:** Siqiong Jiang, Song Mao.

**Validation:** Song Mao, Liangxia Wu.

**Visualization:** Liangxia Wu.

**Writing – original draft:** Li Fang, Jiaoru Pei, and Siqiong Jiang.

**Writing – review & editing:** Li Fang, Jiaoru Pei, and Siqiong Jiang.
